# Relieving the burden of intensive labeling for stress monitoring in the wild by using semi-supervised learning

**DOI:** 10.3389/fpsyg.2023.1293513

**Published:** 2024-01-05

**Authors:** Osman Tugay Başaran, Yekta Said Can, Elisabeth André, Cem Ersoy

**Affiliations:** ^1^Computer and Communication Systems (CCS) Labs, Telecommunication Networks Group (TKN), Department of Electrical Engineering and Computer Science, Technische Universität Berlin, Berlin, Germany; ^2^Faculty of Applied Computer Science, Institute of Computer Science, Universität Augsburg, Augsburg, Germany; ^3^NETLAB Research Laboratory, Department of Computer Engineering, Bogazici University, Istanbul, Turkey

**Keywords:** mental stress, psychophysiological, electrodermal activity, CNN-LSTM, label propagation, deep autoencoder, emotion regulation, DBSCAN

## Abstract

Stress, a natural process affecting individuals' wellbeing, has a profound impact on overall quality of life. Researchers from diverse fields employ various technologies and methodologies to investigate it and alleviate the negative effects of this phenomenon. Wearable devices, such as smart bands, capture physiological data, including heart rate variability, motions, and electrodermal activity, enabling stress level monitoring through machine learning models. However, labeling data for model accuracy assessment poses a significant challenge in stress-related research due to incomplete or inaccurate labels provided by individuals in their daily lives. To address this labeling predicament, our study proposes implementing Semi-Supervised Learning (SSL) models. Through comparisons with deep learning-based supervised models and clustering-based unsupervised models, we evaluate the performance of our SSL models. Our experiments show that our SSL models achieve 77% accuracy with a classifier trained on an augmented dataset prepared using the label propagation (LP) algorithm. Additionally, our deep autoencoder network achieves 76% accuracy. These results highlight the superiority of SSL models over unsupervised learning techniques and their comparable performance to supervised learning models, even with limited labeled data. By relieving the burden of labeling in daily life stress recognition, our study advances stress-related research, recognizing stress as a natural process rather than a disease. This facilitates the development of more efficient and accurate stress monitoring methods in the wild.

## 1 Introduction

Stress, defined by the World Health Organization (WHO), has emerged as the health epidemic of the 21st century (Fink, 2009). Factors such as traumatic experiences, family issues, workplace challenges, economic concerns, and the recent COVID-19 pandemic have significantly increased stress levels in society, impairing vital processes such as decision-making, social interactions, and mental wellbeing. In the field of clinical psychiatry, extensive studies are being conducted to diagnose and treat this problem (Maercker et al., 2013). Concurrently, computer scientists are developing smart sensor technologies and machine learning algorithms for stress recognition. Previous research has explored various signals, including electroencephalogram (EEG) (Kalas and Momin, 2016), electrocardiogram (ECG), electromyography (EMG) (Cho et al., 2019), combined ECG and galvanic skin response (GSR) (Sriramprakash et al., 2017), smartphone sensors and usage data (Garcia-Ceja et al., 2016), and Photoplethysmogram (PPG) (Can et al., 2020a), to detect stress levels. Wearable smart devices enable the direct collection of physiological data related to stress, such as movements, brain activity, muscle tension, heart rate, and electrodermal activity. Other studies investigate image, video, and speech data using cameras and microphones, but privacy concerns and compatibility issues with in-the-wild environments limit the use of these devices for daily life monitoring.

Initially, stress monitoring research took place in controlled environments such as laboratories or offices, where subjects could be continuously monitored, and context was always known. However, researchers realized that stress experienced in these artificial settings differs from the stress encountered in daily life, which holds greater significance to individuals (Picard, 2016). Daily life stress reflects the complexities and nuances of real-world experiences. It arises from authentic situations that people encounter in their day-to-day lives, making it more ecologically valid. In contrast, laboratory-induced stress may not fully replicate the natural stressors individuals encounter outside the controlled environment.

Furthermore, since the participants know that the inducer does not occur in their lives and affect their life directly in most cases, their reaction level might be lower than they give to a daily life stressor. While researchers strive to design tasks that simulate real-life stressors, the artificial nature of the laboratory setting may limit the generalizability of findings to everyday life. As a result, the focus of research shifted toward studying stress in unrestricted real-life environments. Nevertheless, tracking subjects' physiological data continuously in their daily lives and labeling this data for specific periods present new challenges. With limited knowledge of the context, researchers heavily rely on self-reports from participants as labels (ground truth) to train machine learning algorithms for classification or regression tasks.

Despite the significant effort and resources required for labeling, it is crucial to acknowledge that the success of supervised learning models hinges on the quality of the labels in the dataset. Hence, the objective is to develop techniques that alleviate the labeling burden without compromising performance. Semi-supervised learning (SSL) architectures have been proposed precisely for this purpose, providing a viable solution for problems with sparsely labeled data that is expensive and challenging to collect. SSL leverages both supervised and unsupervised learning approaches, making mathematical assumptions about the dataset's distribution with a small amount of labeled data and a sufficient quantity of unlabeled data. Inductive and transductive methods are employed under the SSL umbrella to establish these assumptions. Given the nature of our problem and data, we identified graph-based and deep neural network-based SSL solutions as promising avenues, particularly considering the underexplored potential of SSL techniques with multi-sensor physiological raw data in the literature.

In this study, we applied semi-supervised learning architectures for stress recognition in daily life scenarios. We utilized a comprehensive physiological dataset (Can et al., 2020b) recorded from individuals in unrestricted environments. This dataset includes continuous tracking of 14 different subjects for 1 week, using the Empatica E4 smart band to collect electrodermal activity (EDA), blood volume pressure (BVP), skin temperature (ST), and 3D accelerometer data. By selecting this dataset, we aimed to investigate the binary stress classification problem (stress class vs. non-stress class) that occurs in subjects' daily lives, without the constraints of a laboratory setting. We explored both graph-based and deep neural network-based SSL methods in the stress recognition context. To demonstrate the performance benefits of reducing the labeling burden using SSL models, we compared them with state-of-the-art supervised/unsupervised machine learning and deep learning models. This study represents a robust implementation of SSL models with raw multi-modal physiological sensor data collected in unrestricted daily life scenarios for stress recognition.

## 2 Related work

In a general perspective, stress recognition studies can be classified under restricted environments (i.e., laboratory, office) and unrestricted daily life environments (Can et al., 2019a). Studies were mostly carried out in a laboratory setting. Firstly, traditional classifiers were explored with handcrafted features (Mozos et al., 2017; Can et al., 2019b; Garg et al., 2021). Then, state-of-the-art deep learning architectures such as regular CNNs (Ghosh et al., 2022) and (Gil-Martin et al., 2022), Hybrid CNNs (Rashid et al., 2021), the combination of CNN and LSTM algorithms (Rastgoo et al., 2019), 1D-CNNs, and LSTM (Feng et al., 2023) were applied in these laboratory studies and promising results of around 90% stress recognition accuracy were obtained.

Stress data collected in the laboratory environment is different from daily life. The primary objective of laboratory studies is to aid individuals in effectively managing their stress levels during their daily life routines. However, recognizing stress in the wild is more challenging because of unlimited movements, unknown context, lack of golden standard ground truth (only self-reports), and low data quality of unobtrusive devices that are suitable for daily life usage. Due to these issues, daily life stress recognition accuracies are generally lower. The research on daily stress recognition began by employing conventional machine learning algorithms such as k Nearest Neighbor (kNN), Support Vector Machine (SVM), and Random Forest, as stated in Inoue (2018), cStress, and Gjoreski et al. (2016). For instance, Smets et al. (2018) curated a large multimodal dataset comprising electrocardiogram (ECG), EDA, Skin Temperature, and Acceleration data from 1,002 participants. They applied the Random Forest classifier and obtained an F1 score of 0.43. Subsequently, with the emergence and widespread adoption of neural networks in the field of machine learning, stress recognition studies also incorporated them (Can et al., 2020b). However, the accuracy achieved by the aforementioned studies in stress level recognition was ~70%, still considerably distant from being highly accurate (refer to Table 1). To enhance stress recognition accuracy, Inoue (2018) introduced contextual information such as step count, sleep duration, and calorie usage to physiological signal data. They achieved an accuracy of 85.40% in recognizing perceived stress levels using the decision tree classifier. Similarly, Gjoreski et al. (2016) improved daily life stress recognition accuracy from 76 to 92% by incorporating activity context and utilizing a Random Forest classifier. Especially deep learning algorithms rely on large amounts of labeled data. However, for long-term daily life monitoring studies, it is challenging to collect complete and high-quality labels.

**Table 1:** Literature review on stress detection.

**References**	**Learning model**	**Stress signal**	**Dataset**	**Environment**	**Unobtrusive**	**Number of participants**	**Duration (p/ part.)**	**Method**	**Accuracy**
Rashid et al. (2021)	Supervised L.	Photoplethysmography (PPG)	WESAD	Laboratory	Yes	15	1 h	Hybrid CNN	88.56
Lai et al. (2021)	Supervised L.	Electrocardiogram (ECG), respiratory, Electromyography (EMG), EDA, PPG, ACC, skin temperature	WESAD	Laboratory	No	15	1 h	Multi layer perceptron (MLP)	97.75
Ghosh et al. (2022)	Supervised L.	Electrocardiogram (ECG), respiratory, Electromyography (EMG), EDA, PPG, ACC, skin temperature	WESAD	Laboratory	No	15	1 h	Convolutional neural network (CNN)	94.8
Garg et al. (2021)	Supervised L.	Electrocardiogram (ECG), respiratory, Electromyography (EMG), EDA, PPG, ACC, skin temperature	WESAD	Laboratory	No	15	1 h	k-NN, linear discriminant analysis, random forest (RF), AdaBoost, and Support Vector Machine (SVM)	83.34 F1
Gil-Martin et al. (2022)	Supervised L.	Electrocardiogram (ECG), respiratory, Electromyography (EMG), EDA, PPG, ACC, skin temperature	WESAD	Laboratory	No	15	1 h	CNN	96.6
Rastgoo et al. (2019)	Supervised L.	ECG	Driving_Simulation	Laboratory	No	24	45 min	CNN-LSTM	92.8
Dalmeida and Masala (2021)	Supervised L.	ECG, EMG, GSR, HR, and respiratory	Driving_Simulation	Laboratory	No	24	45 min	kNN, SVM, MLP, RF	0.79 F1
Feng et al. (2023)	Supervised L.	PPG, ECG, EDA, EMG, ACC	WESAD	Laboratory	No	15	1 h	1D-CNN, LSTM	94.9
Hovsepian et al. (2015)	Supervised L.	ECG, respiratory, accelerometer	cStress	Daily Life	No	20	1 week	Support Vector Machine (SVM)	72
Can et al. (2020b)	Supervised L.	PPG, EDA, Skin temperature	Lab_to_daily	Daily life	Yes	14	1 week	MLP, random forest, SVM, logistic regression	73
Can and André (2023)	Supervised L.	PPG, EDA, Skin Temperature	Lab_to_daily	Daily life	Yes	14	1 week	LSTM, GRU, CNN-LSTM, 1D CNN	95
Smets et al. (2018)	Supervised L.	ECG, EDA, ACC Skin temperature	SWEET	Daily life	Yes	1002	5 days	Random Forest	0.43 F1
Inoue (2018)	Supervised L.	Heart rate, step count, Sleep and calories	Local	Daily Life	Yes	10	10 days	kNN, SVM and decision tree	85.40
Gjoreski et al. (2016)	Supervised L.	PPG, skin temperature, EDA, HeartRate (with context info)	Local	Daily life	Yes	5	11 days	Random forest	92
Yu and Sano (2022)	Semi Supervised L.	ECG, EDA, ACC Skin temperature	SMILE and TILES	Daily Life	Yes	45 and 212	5 days	LSTM-AE	63.44 (max.)
Our Work (2023)	Semi-Supervised L.	EDA, BVP ACC, ST	Lab_to_daily	Daily life	Yes	14	1 week	Label propagation Deep autoencoder based SSL	77

After recognizing the importance of labeling challenges when creating stress detection systems, semi-supervised architectures are proposed to relieve this burden. Yu and Sano (2022) developed a sequence-to-sequence LSTM auto-encoder (LSTM-AE) and combined it with data augmentation and consistency regularization techniques. They applied their semi-supervised learning approach to SWEET (Smets et al., 2018) and TILES (Mundnich et al., 2020) datasets and obtained a maximum of 0.65 accuracy which is far from a robust performance. They extracted handcrafted features from in-the-wild physiological data and applied a three-layer LSTM in a semi-supervised setting. They obtained accuracies from 0.58 to 0.65 in binary-level stress classification. However, using raw data and systems instead of windowing and feature extraction increases the amount of data, and RNN variants can learn better and result in higher accuracies. The model constructed with the sequence-to-sequence approach might give more weight or importance to recent inputs in the sequence, potentially neglecting or downplaying earlier information. This bias can affect the model's ability to capture long-term dependencies or make accurate predictions that require a broader context. On the other hand, the consistency regularization that the authors use aims to encourage the model's predictions to be consistent across different views or perturbations of the same input. This method can help improve generalization and robustness. However, if the regularization enforces strong consistency requirements that are not appropriate for the underlying data distribution, the model might become biased toward certain patterns or fail to capture certain variations.

The same authors tested semi-supervised learning systems for both stress and activity recognition tasks (Yu and Sano, 2023). After noise removal and windowing, they used an autoencoder for feature extraction purposes. They reported a maximum accuracy of 63.44 in the binary stress recognition task which is just above the random baseline. Stress recognition is still a new field for SSL architectures in the wild and in terms of performance, there is still room for improvement. Recognizing this new research opportunity, we wanted to carry out a robust study that works with SSL architectures and raw, multi-modal daily life data. We explored both graph and deep neural network-based semi-supervised learning algorithms in the stress recognition context and compare their performance with unsupervised and supervised learning algorithms.

## 3 Data description

Can et al. (2020b) conducted a daily life experiment involving 14 university students aged between 20 and 25, consisting of nine male and five female participants. Each participant was provided with an Empatica E4 smart band to wear for 1 week. They were instructed to wear the smart bands for 12 h a day, from 9 a.m. to 9 p.m., as part of their daily routine. The days on which they wore the smart bands were not necessarily consecutive. The researchers chose to use an online version of the Perceived Stress Scale (PSS-5) questionnaire for self-reporting. The PSS-5 questionnaire is a shortened version of the PSS-14 and has been found to be highly correlated with it (Cohen et al., 1983). It is also considered suitable for use in ambulatory settings (Plarre et al., 2011). The PSS-5 consists of six Likert scale questions related to five emotions, with two of them being positive (happy and cheerful) and three of them (anger, sadness, and frustration) being negative. To calculate the total stress score, the scores for positive emotions are reversed, while the scores for negative emotions are used directly. For instance, if a participant rates their happiness as 6 on a scale of 1–6 (extremely happy), it is evaluated as 1 since happiness and cheerfulness are inversely related to stress levels. Conversely, the scores for anger, sadness, and frustration are added directly when calculating the total stress score (see Equation 1) (Can et al., 2020b).


(1) $$\begin{array}{l} PercStress=\left(7-H_{i}\right)+\left(7-C_{i}\right)+A_{i}+S_{i}+F_{i} \end{array}$$

where *H*_*i*_, happiness score; *C*_*i*_, cheerfulness score; *A*_*i*_, anger score; *S*_*i*_, sadness score; and *F*_*i*_, frustration score. Total scores can change from 0 to 30. Scores ranging from 0 to 15 (midpoint) are considered as low perceived stress and scores ranging from 15 to 30 are considered as high perceived stress. They divided the scores by adapting the 3-class division of PSS-14 (PSS, 2020). Participants were instructed to complete the questionnaire every 3 h, referred to as a “session.” To ensure the collection of self-reports, reminder emails containing questionnaire links were sent at the end of each session for seven consecutive days while participants were wearing the wristband. Specifically, participants were reminded to fill in the PSS-5 questionnaire at 12 p.m., 3 p.m., 6 p.m., and 9 p.m. throughout the seven-day period. The survey app, accessible on both desktop and mobile browsers, was utilized to deliver the questionnaire to participants. The questionnaire link was provided to participants via email. In total, the researchers obtained 989 h of physiological data and 332 self-reports. Some sessions had missing Ecological Momentary Assessments (EMAs), totaling 60 missing EMAs, and the corresponding physiological data for those sessions were excluded. The synchronized raw data and extracted features can be accessed through the provided link: https://github.com/ysaidcan/mood_aware_emotion_recog.

The distribution of self-reported ground truth labels in the daily life dataset was imbalanced, with 73% of the data belonging to the relaxed class and the remaining 27% to the stressed class. Empatica E4 smart band collected electrodermal activity (EDA) with a 4 Hz sampling rate, blood volume pressure (BVP) at 64 Hz, skin temperature (ST) at 4 Hz, and 3D accelerometer data at a 32 Hz sampling rate. The data were averaged for 1-s intervals to equalize different sampling rates for synchronization. To address this issue, we employed a commonly used technique for imbalanced datasets, which involves randomly undersampling the majority class (relaxed class) to obtain a balanced representation of the data (Kotsiantis et al., 2006). In this method, the majority of class instances are randomly deleted and the distribution of two classes becomes balanced.

## 4 Proposed semi-supervised learning architectures and benchmarking

**Figure 1:**
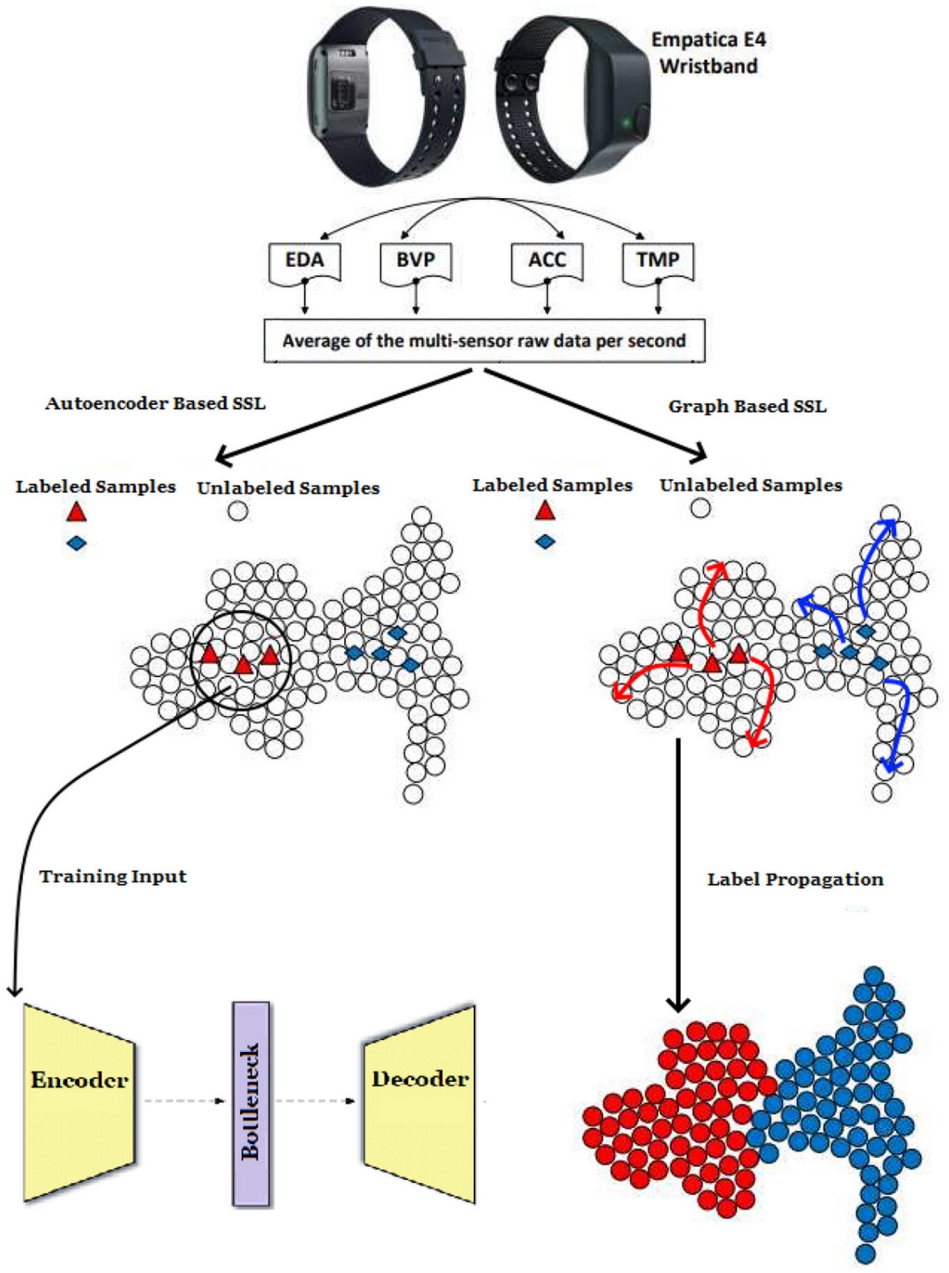
The block diagram of the stress detection system with two different semi-supervised learning models.

**Figure 2:**
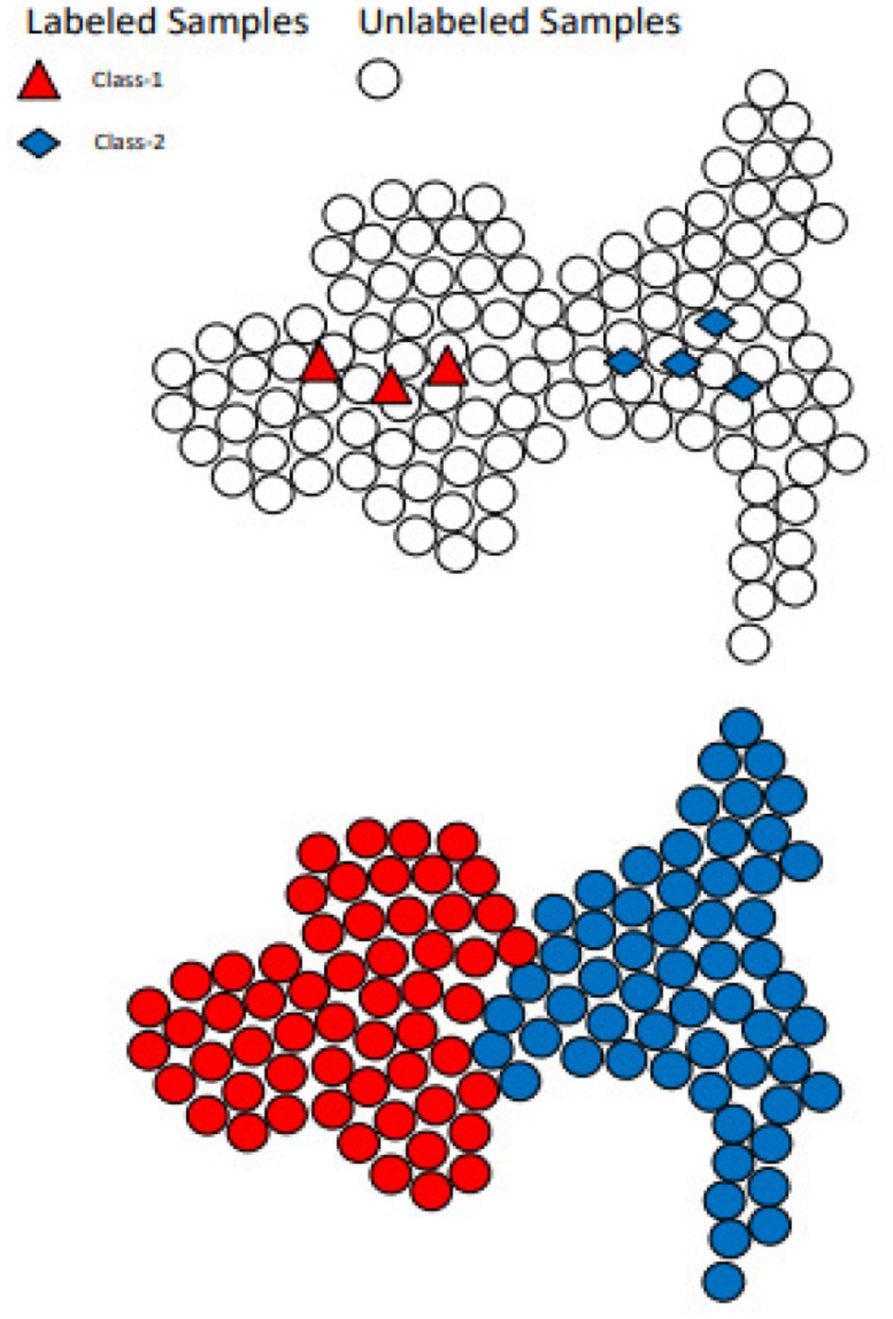
Label propagation mechanism is shown through labeled data samples.

An overview of the approaches employed in our research is depicted in Figure 1. Fusion techniques are utilized to prepare the multi-sensor raw physiological data, which is subsequently employed as input in our Label Propagation and Deep Autoencoder models. The operational principle of the LP algorithm is illustrated in Figure 2. This section will provide a detailed explanation of our Label Propagation and Deep Autoencoder-based architectures. In Sections 4.3 and 4.4, we implemented LSTM, CNN-LSTM, and Clustering algorithms to assess the effectiveness of the proposed SSL models. We also created an open repository with the codes and the link is as follows: https://github.com/basarantugay/ssl-stress-paper.

### 4.1 SSL via label propagation unit

Label Propagation (LP) is an SSL technique grounded in graph theory. In this approach, nodes represent data samples, and edges symbolize the similarity between nodes. Propagation, facilitated through nodes with known labels, allows unlabeled nodes to adopt labels akin to those of the labeled nodes (Bengio et al., 2006).

#### 4.1.1 Theoretical formulation and preliminaries

The algorithm was developed by Zhu and Ghahramani (2002). The mathematical formulation can be expressed as: *Labeled data:*


(2) $$\begin{array}{l} \left(x_{1},y_{1}\right)...\left(x_{n},y_{n}\right),where\;Y_{N}=\left(y_{1}...y_{n}\right)\in \left\{1...C\right\}, \end{array}$$

*x*_*n*_ refers to data points, and *Y*_*N*_ refers to the labels for the different classes available in our dataset. While *C* expresses the number of classes, we assumed that there are samples from each class in the dataset. *Unlabeled data:*


(3) $$\begin{array}{l} \left(x_{n+1},y_{n+1}\right)...\left(x_{n+t},y_{n+t}\right),where\;Y_{T}=\left(y_{n+1}...y_{n+t}\right), \end{array}$$

*Entire dataset:*


(4) $$\begin{array}{l} X=\left\{x_{1}...x_{l+t}\right\}\in R^{D}. \end{array}$$

After the dataset is defined, the ultimate goal is to estimate *Y*_*T*_ using *X* and *Y*_*N*_. A fully connected graph infrastructure has been designed to solve the problem. While each data sample in the dataset represents a node, the relationship between nodes is weighted thanks to the calculated Euclidean distance. The weights can be expressed mathematically as:


(5) $$\begin{array}{l} w_{ij}=exp\left(-\frac{d_{ij}^{2}}{\sigma^{2}}\right) \end{array}$$

Labels are disseminated to all unlabeled data samples based on the edges determined by weights. σ is employed as a control parameter for weight calculation. The probabilistic transition matrix is utilized to accurately assign labels. Consequently, labels of nodes can be updated along edges with higher weights. The matrix can be expressed as:


(6) $$\begin{array}{l} T_{ij}=P(j\to i)=\left(\frac{w_{ij}}{\sum_{k=1}^{l+t}w_{kj}}\right) \end{array}$$

The probability of transition from node j to node i is calculated through the *T*_*ij*_ matrix defined in (*l*+*t*)*x*(*l*+*t*). As can be seen from the formula, this calculation changes according to the weight of the edges between the nodes. Matrix *Y*, whose size is defined as (*l*+*t*)*xC* depending on the number of classes, keeps the label probabilities of the nodes. The working principle of the algorithm consists of three basic steps:

1. All nodes propagate labels using the probabilistic transition matrix *Y*.
2. *Y* matrix rows are normalized to provide class probability interpretation.
3. Run the algorithm from step 2 until *Y* converges.

#### 4.1.2 Algorithm implementation

The algorithm is implemented using the sci-kit-learn library (Pedregosa et al., 2011). The strategy followed during implementation is shown in Algorithm 1.

**Algorithm 1:** SSL via label propagation.

1: Split the test data. (Test Size = 30%)
2: Split the training dataset into labeled and unlabeled data points.
3: Predict the labels of unlabeled samples with the label propagation algorithm.
4: The pseudo-labels, which are the outputs of the Label Propagation algorithm, are replaced with unlabeled samples in the training dataset.
5: Training the classifier with the new augmented dataset of labeled and pseudo-labeled samples.
6: Use this model to predict test data.

#### 4.1.3 Hyper-parameter optimization

The LP algorithm has two different kernel functions. Therefore, before starting the performance evaluation, we experimented with these kernel functions. The best performance of the LP algorithm was obtained by choosing the correct kernel function. LP algorithm has kNN and RBF options as its kernel. Tables 2, 3 show the performance metrics obtained using these two kernels. LP algorithm with kNN kernel has high precision scores for both classes. f-Measure is also acceptable (0.9 for the non-stress class and 0.7 for the stress class).

**Table 2:** Performance of the label propagation algorithm for creating pseudolabels (selected LP Kernel = k-NN).

**Class**	**Precision**	**Recall**	**f-Measure**
Non-stress (%5 labeled data)	0.53	0.60	0.58
Stress (%5 labeled data)	0.56	0.47	0.49
Non-stress (%10 labeled data)	0.65	0.75	0.67
Stress (%10 labeled data)	0.69	0.52	0.56
Non-stress (%15 labeled data)	0.74	0.86	0.82
Stress (%15 labeled data)	0.78	0.64	0.67
Non-Stress (%20 labeled data)	0.86	0.99	0.92
Stress (%20 labeled data)	0.97	0.72	0.75
Macro average	0.92	0.86	0.84
Weighted average	0.89	0.92	0.88

**Table 3:** Performance of the label propagation algorithm for creating pseudolabels (selected LP kernel = RBF).

**Class**	**Precision**	**Recall**	**f-Measure**
Non-Stress	0.86	0.85	0.83
Stress	0.88	0.62	0.68
Macro average	0.87	0.74	0.76
Weighted average	0.86	0.80	0.79

The key point to note is that the original dataset is partially imbalanced, with the non-stress class value count being three times the value count (or the number of samples) of the stress class (almost 1.5 million class-0 and 0.5 million class-1 samples). Consequently, achieving a high f1-score for class-0 is quite natural. However, we still obtained a satisfactory f1 score of 0.75 for class-1. Nevertheless, the performance results of the LP algorithm with the RBF kernel are lower than the kNN kernel version. Upon reviewing Table 3, it is evident that f-measure and recall scores are lower, especially in the non-stress class where the success of the RBF kernel function has decreased. Considering the performance metrics, it has been confirmed that using kNN as a kernel function in the LP algorithm yields better results.

Now, we have new labels successfully classified by the LP algorithm. After adding these observations to the training data (by replacing the unlabeled data with the new predictions) on which we have moderate confidence, these are referred to as pseudo-labeled in contrast to labeled data. Subsequently, we trained this new (augmented) dataset using different classifiers and employed these models to predict the accuracy of the test data, which comprises 651,600 samples.

After tuning the kernel parameter of the label propagation algorithm, experiments were conducted with various classifiers. The performance comparison among these classifiers was based on examining the accuracy score. While the performance results of classifiers such as kNN, Naive Bayes, Logistic Regression, Decision Tree, and Random Forest are closely aligned, the random forest classifier yielded the highest accuracy score.

Specifically, we compared the performance of classifiers before the hyper-parameter tuning stage and then selected the classifier that exhibited the best performance metrics for the hyper-parameter tuning process. In this case, the Random Forest (RF) Classifier had already demonstrated superior performance even before the hyper-parameter tuning stage. With a relatively small set of labeled and unlabeled datasets, assisted by the LP algorithm, we achieved an acceptable accuracy of 75% on this test set.

After this stage, hyper-parameter tuning was performed to increase the performance of the random forest classifier. The RF algorithm consists of many parameters such as criterion, min_samples_split, min_samples_leaf, min_weight_fraction_leaf, max_features, max_leaf_nodes, min_impurity_decrease, bootstrap, oob_score, n_jobs, verbose, warm_start, class_weight. We used *max_depth* and *n_estimators* parameters in the hyper-parameter tuning process. The parameters max_depth and n_estimators are directly related to the learning ability of the model due to their impact on the complexity and capacity of the ensemble. By controlling the max depth, you can limit the complexity of the trees and prevent them from memorizing noise or specific instances in the training data. Increasing the number of trees improves the model's ability to capture diverse patterns and reduces the variance of the predictions. With more trees, the random forest becomes more robust and less sensitive to the idiosyncrasies of individual trees. This helps stabilize predictions, enhance generalization, and reduce overfitting. Due to the competencies of these two parameters, they are preferred over other existing parameters, contributing to an overall enhancement of classification performance. The accuracy scores of the Random Forest (RF) classifier were obtained for parameter values that vary relative to each other, and the results are shared in Table 4. Three different forest scenarios (100, 300, 500) were examined for increasing the maximum depth of tree values. Excessive increase in the *max_depth* parameter can cause the model to overfit, a situation that requires attention. In the case of the random forest classifier, increasing the *max_depth* parameter significantly can lead to overfitting, as it determines the maximum depth of each decision tree in the ensemble. Excessive *max_depth* values can result in trees that are too complex, fitting the training data closely, including noise and random fluctuations. It can lead to memorizing or overfitting individual instances in the training data, sacrificing generalization for specificity. Moreover, higher *max_depth* values can create more complex decision boundaries, making the model less interpretable and more prone to overfitting. Hence, careful tuning of the *max_depth* parameter is essential, and in our experiments, it was observed that the model overfits the training data for values of *max_depth* >20, which is why it was constrained to *max_depth=20*.

**Table 4:** RF classifier's accuracy results for variable *max_depth* and *n_estimators* parameters.

**max_depth**	***n*** **_estimators**		
	**100**	**300**	**500**
2	75.23	75.23	75.23
4	75.49	75.49	75.49
6	75.78	75.78	75.78
8	76.00	75.99	75.99
10	76.11	76.11	76.11
15	76.49	76.50	76.50
20	76.86	**76.88**	76.86

The bold values indicate the highest accuracy performance.

Increasing the number of estimators in the Random Forest ensemble involves incorporating a larger collection of individual decision trees. However, higher values can escalate the model's computational complexity, so it needs to be controlled to prevent excessively long training times. Evaluation of accuracy scores revealed that the best performance was achieved with *max_depth=20* and *n_estimators=300*. Beyond this point, further increasing the *n_estimators* did not significantly improve the model's performance. In summary, the random forest model consisting of 300 trees with a depth of 20 units demonstrated the best overall performance.

**Figure 3:**
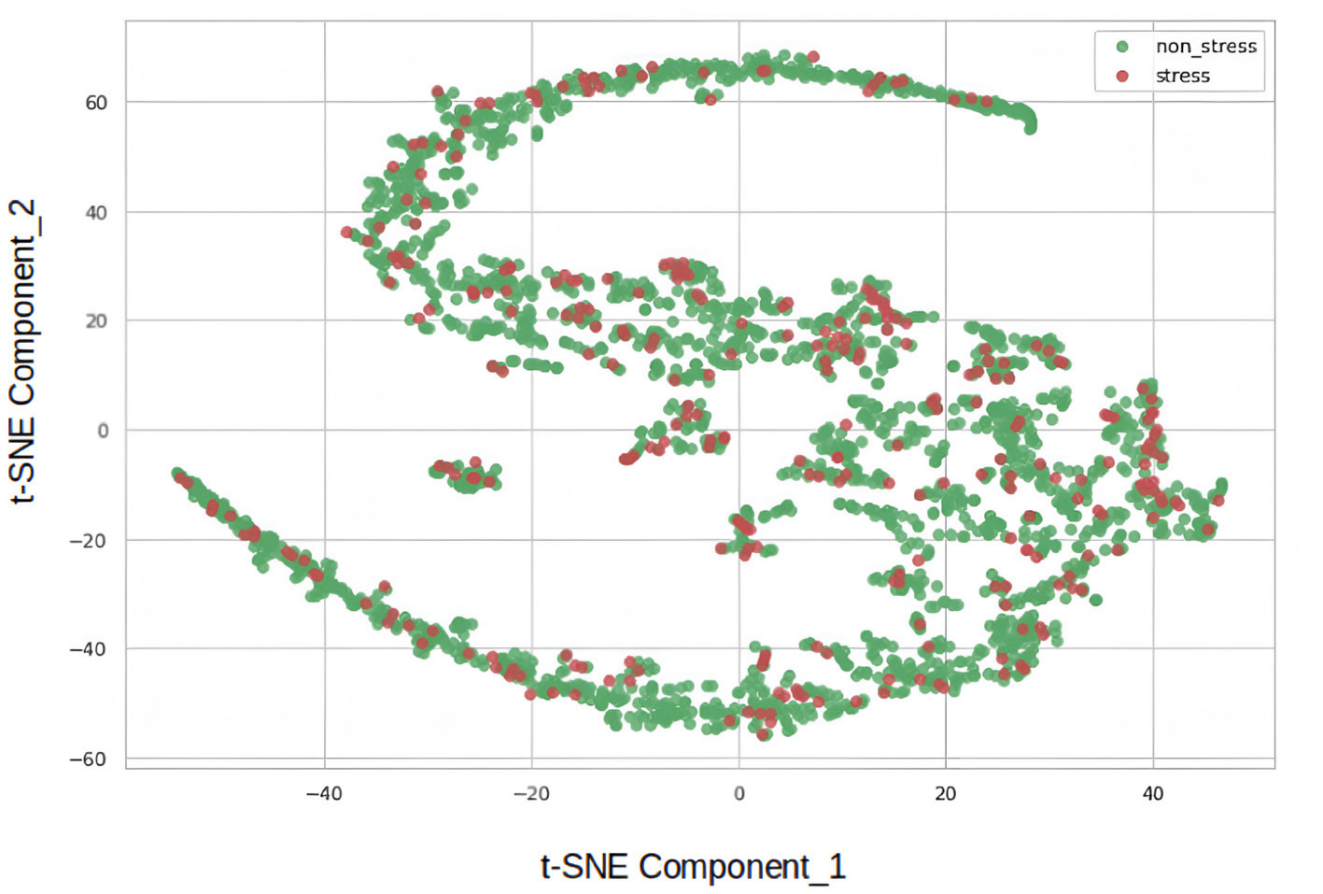
Visualization of stress and non-stress classes via t-SNE.

Moreover, the underperformance of the simple autoencoder model prompted us to experiment with a deep autoencoder design. Prior to the hyper-parameter tuning stages, visualizing the data using the t-Distributed Stochastic Neighbor Embedding (t-SNE) nonlinear statistical method was deemed useful. The t-SNE algorithm, developed in 2008 (Van Der Maaten and Hinton, 2008), reduced the dimensions of the data, providing a two-component representation. Figure 3 depicts the result, where red dots represent the stress class, and green dots belong to the non-stress class. The close proximity of samples from both classes suggests that the dataset poses a significant challenge for simple models.

As the next step, evaluating how close we are to the performance of the label propagation algorithm by training a more robust classifier becomes crucial. For this reason, experiments were conducted with the MLP classifier. Initially, the classifier was trained with its default parameters, and performance results were obtained. The parameters used during the initial training are:

- Hidden layer size = (100,), Activation F. = ReLU
- Solver = Adam, Alpha = 0.0001, Learning R. = Constant

The performance results of the MLP classifier, trained using the aforementioned parameters, are presented in Table 5. Surprisingly, the basic MLP Classifier without parameter tuning outperformed the RF Classifier in terms of maximum performance. In light of this, it becomes essential to assess the results by scrutinizing a more comprehensive parameter grid. Hyperparameter tuning was conducted across a broad parameter space, and the parameters yielding the best model estimation were obtained using the sci-kit-learn library's GridSearchCV tool. The best parameters were identified after hyperparameter tuning within the parameter space outlined in Table 6:

- Hidden Layer Size = (50, 100, 50), Activation F. = tanh
- Solver = Adam, Alpha = 0.0001, Learning R. = Adaptive

**Table 5:** Classification report of MLP classifier (default parameters).

**Class**	**Precision**	**Recall**	**f-Measure**
Non-Stress	0.65	0.73	0.69
Stress	0.69	0.61	0.65
Accuracy	0.68		

**Table 6:** Parameter grid via GridSearchCV.

**Parameters**	**Parameter values**
Hidden layer size	[(50, 50, 50), (50, 100, 50), (100,)]
Activation function	[“lbfgs,” “sgd,” “adam”]
Solver	[“logistic,” “tanh,” “relu”]
Alpha	[0.00001, 0.0001, 0.05]
Learning rate	[“constant,” “invscaling,” “adaptive”]

### 4.2 SSL via deep autoencoder unit

Autoencoder studies were initially published in 1986 (Rumelhart and McClelland, 1987). These studies began with the perspective of unsupervised learning to comprehend the internal representation of the data. The input, encoded through a neural network, is then reconstructed, aiming to extract the informative parts of the data.

#### 4.2.1 Theoretical formulation and preliminaries

Autoencoder was expressed mathematically by Baldi (2012). The encoder and decoder functions can be expressed as:


(7) $$\begin{array}{l} Y:{\mathbb{R}}^{n}\to {\mathbb{R}}^{p}\left(encoder\right), \end{array}$$

(8) $$\begin{array}{l} Z:{\mathbb{R}}^{p}\to {\mathbb{R}}^{n}\left(decoder\right), \end{array}$$

while learning the above functions, it is necessary to consider the following constraint:


(9) $$arg\;min_{Y,Z}E[\Delta (x,Z\;o\;Y(x\;)].$$

Expectation over the distribution of *x* is calculated with the help of operator *E*. Δ operator expresses the reconstruction loss function by calculating the distance between the encoder input and the decoder output.

#### 4.2.2 Algorithm implementation

The autoencoder, serving as an unsupervised learning (UL) technique, was tailored to construct a semi-supervised learning (SSL) architecture in our study. By presenting only a small subset of the non-stress samples to the autoencoder model, the model endeavors to learn the optimal representation of the non-stress class. Subsequently, using the same model, stress samples are generated in a distinct manner from non-stress samples. This enables the autoencoder to effectively distinguish the automatically generated stress samples. The pseudocode is outlined in Algorithm 2.

**Algorithm 2:** SSL via deep autoencoder unit.

1: Create an autoencoder network with input and output layers.
2: Apply Min-Max Normalization.
3: Train autoencoder model with a small amount of non-stress samples.
4: Create a new network consisting of the weights of the trained network (This will create a network of latent representations of non-stress samples.).
5: Predicting raw non-stress and stress samples' hidden representation.
6: Hyper-parameter tuning in parameter space(hidden layer sizes, activation function, solver, alphas, learning rate).
7: Train and validate the classifier with the dataset containing the latent representation with the best parameters.

#### 4.2.3 Hyper-parameter optimization

**Figure 4:**
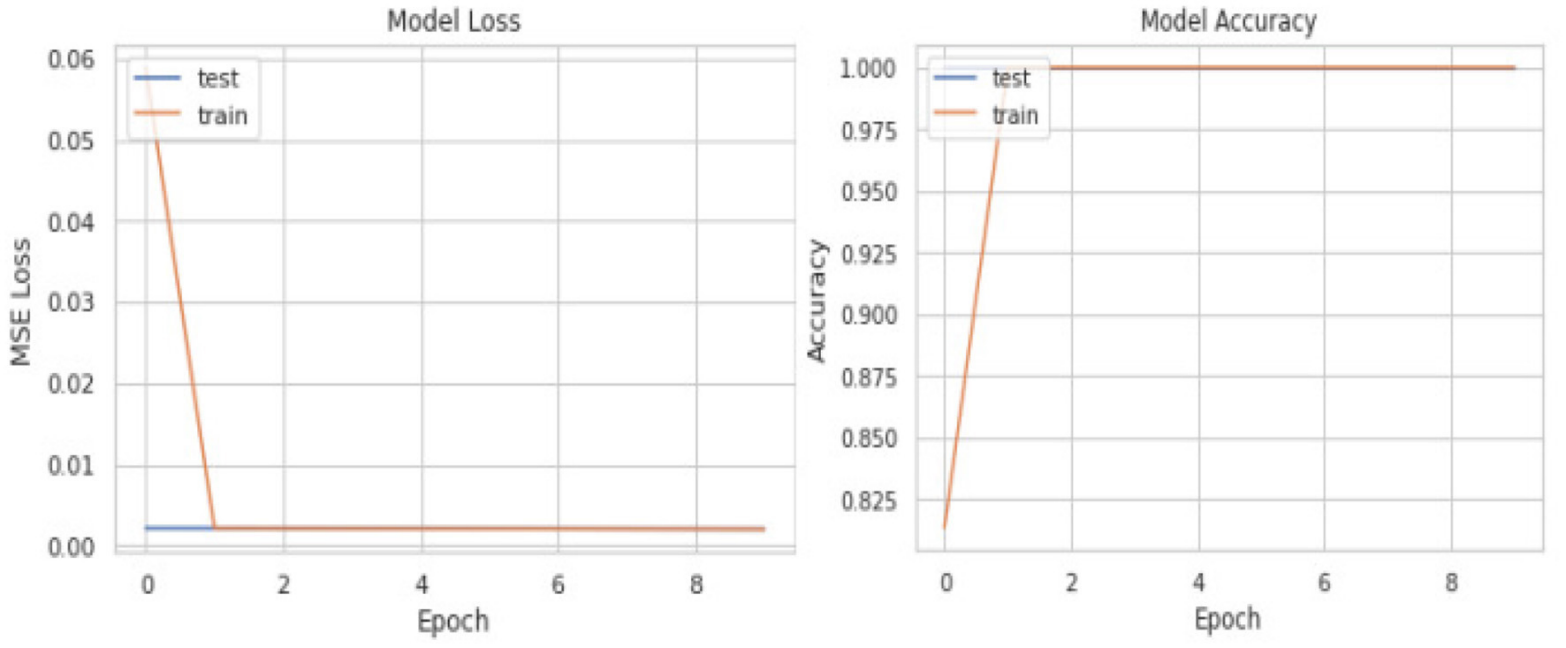
Problematic learning curves of initial model with simple autoencoder where parameters could not be tuned precisely.

Initially, we aimed to explore the results by commencing with a simple model. Table 7 illustrates the design of our initial, less complex autoencoder model. This model lacks batch normalization layers, features fewer dense layers, possesses a bottleneck size much larger than the input size, and has almost half the number of trainable parameters compared to our final model. The learning curves, depicted in Figure 4, clearly indicate that the model is prone to overfitting the training data. Consequently, making predictions based on input reconstruction with such an autoencoder model is likely to yield fallacious and biased results.

**Table 7:** First experiments of deep autoencoder model summary.

**Autoencoder layers**	**Output shape**	**Parameters**
input_1 (InputLayer)	(None, 4)	0
dense (dense)	(None, 100)	500
dense_1 (dense)	(None, 50)	5,050
dense_2 (dense)	(None, 50)	2,550
dense_3 (dense)	(None, 100)	5,100
dense_4 (dense)	(None, 4)	4,004
Total params: 13,604		
Trainable params: 13,604		
Nontrainable params: 0		

Considering the challenges posed by our data, the design of the autoencoder was carefully executed. Attempting to work with a high-dimensional and imbalanced dataset using a single-layer autoencoder proved impractical for effective learning from the data. Consequently, we devised a Deep Autoencoder architecture utilizing stacks of layers, symmetrically created on both the encoder and decoder parts. The encoder and decoder components comprise shallow layers interconnected with a bottleneck.

In an autoencoder, the bottleneck represents the layer or segment of the network with lower dimensionality compared to the input and output layers. This central layer compresses the input data into a condensed representation, crucial for capturing the most salient features or patterns. The bottleneck is pivotal in achieving dimensionality reduction, extracting and representing features, supporting unsupervised learning, noise reduction, and data compression and reconstruction.

We conducted numerous experiments to determine the input nodes of the shallow layers in the encoder. With four features in mind, the dense layer, starting with a hundred nodes, was gradually reduced to three nodes as it approached the bottleneck. The design decision for the bottleneck size is significant; experiments revealed that an excessively large bottleneck results in a network that merely copies the input's low-dimensional representation, while a too narrow bottleneck hampers the network's learning capacity due to significant information loss. Consequently, the number of nodes was systematically reduced from one hundred to three in the encoder part. The encoder layers were designed with a width of 100, 75, 50, 25, and 3 nodes, respectively. In the decoder, which mirrors the encoder, layers were designed incrementally for input reconstruction with 3, 25, 50, 75, and 100 nodes. The gradual reduction and subsequent increase in node width were found to enhance the network's learning ability. The introduction of noise to the encoder side improved learning outcomes.

L1 regularization was employed as a feature selection mechanism, encouraging the autoencoder to focus on informative features while discarding less important ones. Given our high-dimensional dataset, L1 regularization was deemed advantageous, and a regularization value of 0.00001 was selected based on experiments. Mean Squared Error Loss (MSE Loss) was chosen as the error measurement between the actual and reconstructed input.

Activation functions were strategically chosen within the layers, using tanh in the first two encoder layers, tanh in the last two decoder layers, and ReLU for the remaining layers. The mixed design was found to yield superior performance. A deep autoencoder entails numerous stacked layers, making recursive training computationally intensive and prone to overfitting. Batch normalization was incorporated to enhance network reliability, prevent overfitting, and expedite convergence during layer designs. Multiple BatchNorm layers were utilized, particularly beneficial for high-dimensional and imbalanced datasets, contributing to dimensionality reduction, balanced representations, and improved generalization.

During the autoencoder training phase, the *batch_size* was systematically adjusted, with *batch_size=256* yielding the best performance. Adadelta optimizer, a stochastic gradient descent method with an adaptive learning rate, was identified as the most effective optimizer through experimentation. The adaptability of Adadelta without depending on the initial learning rate proved advantageous in the hyper-parameter tuning phase.

**Figure 5:**
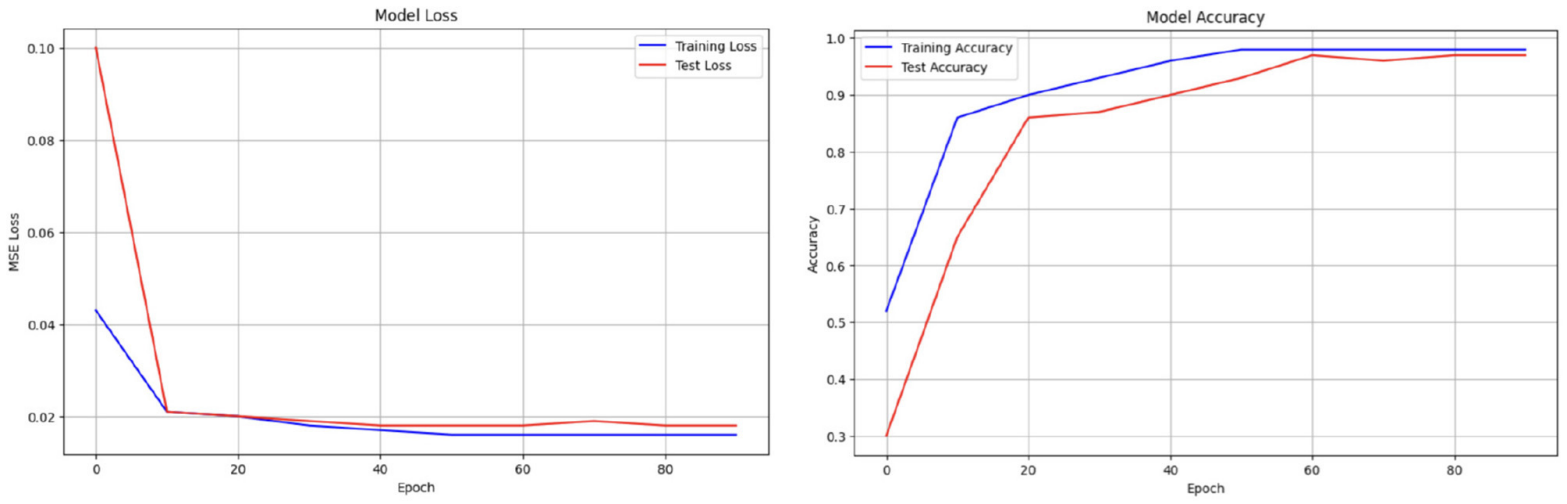
Learning curves of our final best performing deep autoencoder model.

**Figure 6:**
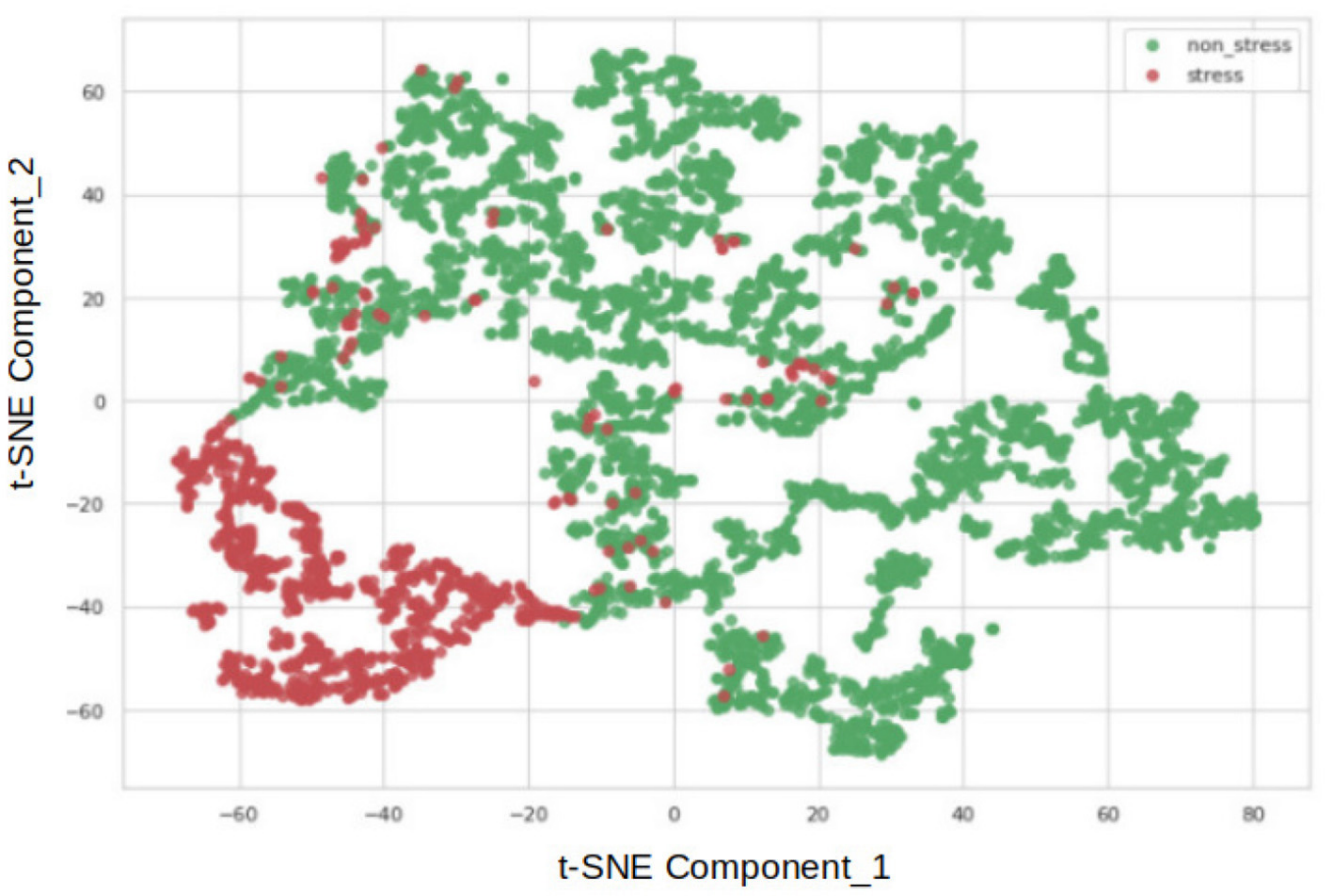
Visualization of stress and nonstress classes via t-SNE by reconstructing the latent representation of non-stress input.

A comprehensive overview of the deep autoencoder model is presented in Table 8. During the design process, decisions were guided by achieving optimal training performance, as evidenced by the examination of loss and accuracy curves in Figure 5. The success of classifying stress and non-stress samples by reconstructing the latent representation of non-stress input is illustrated in Figure 6.

**Table 8:** Final deep autoencoder model summary with parameters.

**Autoencoder layers**	**Output shape**	**Parameters**
input_1 (InputLayer)	(None, 4)	0
dense (dense)	(None, 100)	500
batch_normalization (BatchNorm)	(None, 100)	400
dense_1 (dense)	(None, 75)	7,575
batch_normalization_1 (BatchNorm)	(None, 75)	300
dense_2 (dense)	(None, 50)	3,800
batch_normalization_2 (BatchNorm)	(None, 50)	200
dense_3 (dense)	(None, 25)	1,275
batch_normalization_3 (BatchNorm)	(None, 25)	100
dense_4 (dense)	(None, 3)	78
dense_5 (dense)	(None, 3)	12
batch_normalization_4 (BatchNorm)	(None, 3)	12
dense_6 (dense)	(None, 25)	100
batch_normalization_5 (BatchNorm)	(None, 25)	100
dense_7 (dense)	(None, 50)	1,300
batch_normalization_6 (BatchNorm)	(None, 25)	100
dense_8 (dense)	(None, 75)	3,825
batch_normalization_7 (BatchNorm)	(None, 75)	300
dense_9 (dense)	(None, 100)	7,600
dense_10 (dense)	(None, 4)	404
Total params: 28,081		
Trainable params: 27,275		
Nontrainable params: 806		

Subsequently, the performance of the dataset obtained with the deep autoencoder model was evaluated using various classifiers, starting with a linear classifier. Limited-memory Broyden Fletcher Goldfarb Shanno (L-BFGS) algorithm was initially used as a solver, but the results did not meet expectations, with an overall accuracy of 0.61, slightly above 50%. Given that L-BFGS solver only supports L2 regularization, it imposes a constraint, prompting a shift to the SAGA optimizer. While the performance results with SAGA were marginally better than L-BFGS solver, the overall classifier accuracy increased to around 0.63. Despite this improvement, the expected performance scores were not achieved.

To further evaluate the dataset, an augmented dataset created through the LP algorithm was subjected to a Random Forest (RF) classifier, employing similar parameters (*max_depth=20, n_estimators*). The RF classifier achieved an accuracy score of 0.68. Although this performance appears more successful than the linear classifier, there is still room for enhancement. Notably, when the RF classifier was trained with similar parameters, it was observed that the augmented dataset generated by the label propagation algorithm yielded superior results compared to the dataset created with the deep autoencoder.

### 4.3 Supervised learning—Deep neural networks

In supervised learning, employing networks like LSTM and CNN provides an advantage in terms of feature engineering, allowing direct use of raw data. Consequently, we opted to utilize LSTM and CNN-LSTM deep learning architectures for stress recognition. Given that physiological sensor data is time-series in nature, initiating the design with an LSTM architecture that accommodates sequential input seemed advantageous. Additionally, since features were not pre-extracted from raw data, investigations were conducted on the CNN-LSTM architecture, where CNN layers facilitate superior feature extraction before feeding the data into the LSTM layer. This approach is supported by various studies in the literature (Zhao et al., 2019).

However, it's crucial to address potential biases in LSTM and CNN-LSTM models arising from cross-validation implementation policies, especially when dealing with time-series data. Shuffle splitting during training can lead to biased results, particularly as the LSTM network perceives data sequences both ahead and behind during training. To mitigate this, a time series data split method is employed, avoiding division into different participants (i.e., personalized models are not used) and treating the data as a whole. In training, time series split techniques are applied with *k*-fold cross-validation, where *k* is set to 5, resulting in four splits for training and one split for validation. This choice aligns with previous studies in the field for comparability reasons (Yu and Sano, 2023).

The models were implemented using the PyTorch Deep Learning Tensor Library in a Google Collaboratory Notebook. This approach allows efficient training of the models with the large dataset, utilizing the GPU processing capability of the existing server through the CUDA API. The evaluation of the current NVIDIA Tesla T4 GPU performance and competence determined a maximum initial batch size of 128. Since the problem at hand involves binary classification, hyper-parameter tuning was conducted based on the accuracy score. This decision facilitates easy comparison of the performances of our semi-supervised (SSL) and unsupervised learning (UL) architectures using the accuracy score in the final discussion. The experiments commenced with the LSTM network.

Our goal was to enhance network performance by tuning Hidden Size, Learning Rate, Activation Function, and Batch Normalization parameters in the LSTM and CNN-LSTM models. The Hidden Size parameter, representing the number of features in the hidden state, was fine-tuned by gradually increasing it. For *hidden_size* values < 20, the model exhibited underfitting, resulting in lower accuracy scores. Conversely, for *hidden_size* values >20, the risk of overfitting increased, leading to insufficient accuracy scores. To strike a balance, *hidden_size* was set to 20.

The Learning Rate was set to 0.0001 to balance the trade-off between overshooting the global optimum (with larger learning rates) and reduced learning capacity and longer training times (with smaller learning rates).

Different activation functions were experimented with, and ReLU outperformed others, providing better performance and efficient, fast training by disallowing negative gradients. Similar to the deep autoencoder model, a Batch Normalization layer was integrated into the LSTM network, enhancing its reliability in terms of stability and robustness. Batch Normalization is particularly beneficial for LSTM networks, which are known for capturing long-term dependencies in sequential data.

During training, Binary Cross Entropy (BCE) loss was employed instead of Mean Squared Error (MSE) loss, given the binary classification nature of the problem. The output layer was designed with a sigmoid function, aligning with BCE loss.

Experiments with the CNN-LSTM model followed a similar hyper-parameter tuning strategy based on accuracy metrics. In addition to accuracy, loss curve, f-measure, recall, and precision metrics were concurrently monitored. The maximum batch size, determined during the LSTM network training phase, was maintained at 128 for the CNN-LSTM network, considering the overall network structure.

In the CNN-LSTM network, parameters were separately tuned for the CNN and LSTM parts. Parameters like Kernel Size, Stride, and Hidden Size were tuned for the CNN network, taking into account the dataset dimensions. Kernel Size and Stride were tested in the range of one to three, considering the four attributes in the convolution layer. A kernel size of two provided optimal performance, mitigating overfitting, and a stride of one was set accordingly.

The CNN network's Hidden Size parameter was determined by observing similar findings as in the LSTM model experiments, maintaining consistency with its value in the LSTM network. The LSTM network layers were designed based on the initial LSTM model, with input sizes adjusted according to the output of the CNN network part.

Additionally, 17% of the labeled data (the same percentage used in SSL models) was employed in the LSTM and CNN-LSTM models, ensuring performance measurements on an equivalent amount of data. The detailed results are presented in **Table 12**.

### 4.4 Unsupervised learning—clustering analysis

When assessing our problem and dataset, we decided to employ three different clustering algorithms. Some of these algorithms require predefined parameters such as the number of clusters or the minimum distance between observations. To address the challenge of initializing the cluster number, we utilized a hyper-parameter tuning algorithm, allowing the model to autonomously determine the most suitable cluster number. During the tuning phase, we selected the optimal number of clusters by evaluating the Silhouette Score metric.

K-means, a well-known clustering algorithm, operates by minimizing the average squared distance of samples within the same dense region or cluster. Recognizing that traditional clustering algorithms, like K-means, can be enhanced in areas such as running time, memory management, and processing performance, we explored the Balanced Iterative Reducing and Clustering using Hierarchies (BIRCH) algorithm (Zhang et al., 1996). Additionally, we experimented with the Density-Based Spatial Clustering of Applications with Noise (DBSCAN) algorithm, offering a distinct alternative (Ester et al., 1996).

Given the varied scales of feature units, we standardized the data using the sci-kit-learn *StandardScaler* method before running the three clustering algorithms. Throughout the hyper-parameter tuning phase, we monitored the Silhouette Score to allow the models to autonomously determine the number of clusters. The highest Silhouette Score was achieved with the algorithm selecting two clusters. While we were already aware that our data had two classes, we validated the K-means algorithm's ability to correctly cluster these two classes using the Silhouette Score metric. Similar results were obtained by assessing the Silhouette Score for the BIRCH algorithm. Though the results are closely aligned, it can be inferred that the BIRCH algorithm slightly outperforms in clustering the two classes with minimal difference.

## 5 Experimental results and discussion

### 5.1 Supervised learning architectures performance

In the LSTM model, after completing the hyper-parameter tuning, we present the layers of the finalized model and the number of trainable parameters formed in these layers in Table 9. To assess the benefits of using GPU, we also trained the LSTM model via CPU, and the training times are shared in Table 10. During GPU training, each iteration took 2.2 s, while on the CPU, it took 50 s per iteration. Each iteration involves one forward pass and one backward pass. Training on the GPU resulted in 23 times faster iterations. In the CNN-LSTM model, the layers of the finalized model and the number of trainable parameters formed in these layers are presented in Table 11. The CNN network part of the CNN-LSTM model imposed a substantial load on the CPU, leading to a 70 × longer training time with CPU. However, the training duration of the CNN-LSTM network was lower than that of the LSTM network. Training the CNN-LSTM model with the GPU took 1.1 s per iteration, which is even two times faster than training the LSTM network with a GPU.

**Table 9:** Number of trainable parameters of LSTM network.

**Modules**	**Parameters**
lstm.weight_ih_l0	80
lstm.weight_hh_l0	400
lstm.bias_ih_l0	20
lstm.bias_hh_l0	20
fc.0.weight	12,000,000
fc.0.bias	1,000
fc.2.weight	1,000
fc.2.bias	1,000
fc.3.weight	1,000
fc.3.bias	1
Total trainable params:	12,004,521

**Table 10:** Number of trainable parameters and training time of networks.

**Network**	**Parameters**	**GPU (s)**	**CPU (s)**
LSTM	12,004,521	220	5,000
CNN-LSTM	1,030,753	115	8,000

**Table 11:** Number of trainable parameters of CNN-LSTM network.

**Modules**	**Parameters**
cnn.0.weight	307,200
cnn.0.bias	256
cnn.1.weight	256
cnn.1.bias	256
cnn.3.weight	65,536
cnn.3.bias	128
cnn.4.weight	128
cnn.4.bias	128
lstm.weight_ih_l0	40
lstm.weight_hh_l0	400
lstm.bias_ih_l0	20
lstm.bias_hh_l0	20
fc.0.weight	65,5360
fc.0.bias	256
fc.2.weight	256
fc.2.bias	256
fc.3.weight	256
fc.3.bias	1
Total trainable params:	1,030,753

Considering the performance metrics in Table 12, a more successful result was obtained with the CNN-LSTM network. The results achieved are competitive with those reported in the literature. The CNN-LSTM model exhibited slightly better performance in accuracy, precision, recall, and f-measure compared to the LSTM model. In the CNN-LSTM network, saturation occurred around the 80th iteration, similar to the LSTM model, despite training for 100 epochs. The performance results of the LSTM and the CNN-LSTM models are provided together in Table 12. Although the results were close, the CNN-LSTM model demonstrated improvement. One of the main contributing factors is that, thanks to the CNN layers, more informative features were extracted from the raw data and fed to the LSTM layers, enhancing overall performance. Apart from the performance boost, the CNN-LSTM model provided another valuable output-training using the GPU took significantly less time. Models of this kind facilitate working with large datasets.

**Table 12:** Classification results of LSTM and CNN-LSTM networks: using all labeled data and 17% labeled data.

**Algorithm**	**Accuracy**	**f-Measure**	**Precision**	**Recall**
LSTM	90.38	82.60	83.18	82.01
CNN-LSTM	**91.35**	83.84	85.70	82.09
LSTM (17% labeled data)	72.73	72.52	75.10	70.10
CNN-LSTM (17% labeled data)	74.89	76.32	72.71	80.32

The bold values indicate the highest accuracy performance.

Finally, supervised learning (SL) models were trained with only 17% labeled data for comparison with semi-supervised learning (SSL) models. Examining Table 12 reveals a performance degradation. With less labeled data, the model may have limited exposure to positive instances, leading to a reduced understanding of the positive class and a lower precision score. The decrease in accuracy can be attributed to the lack of diversity and generalization capability in the model's training. In such cases, while SSL models spare us from the burden of labeling, they also achieve promising performance results in scenarios with limited labeled data.

### 5.2 Semi-supervised L. architectures performance

Our Label Propagation algorithm demonstrated impressive precision scores of 86% for class-0 (non-stress) and 97% for class-1 (stress). Examining f-Measure, class-0 achieved a commendable 92%, while class-1 achieved 72%. Subsequently, the classifier trained with the augmented dataset prepared using the label propagation algorithm achieved an accuracy of 77%.

Moving on to the deep autoencoder model, performance results are obtained with the final tuned parameters, as shown in Table 13. In comparison to the simpler autoencoder design, the deep-stacked autoencoder model yielded significantly improved and further improvable results. The final model achieved an 81% precision score for class-0 (non-stress) and 72% for class-1 (stress). Examining f-Measure, a score of 77% was achieved for the non-stress class, and a score of 76% was achieved for the stress class. Ultimately, the classifier trained with the augmented dataset prepared using the deep autoencoder model achieved an accuracy of 76%. These promising results were obtained using a reduced number of labeled samples from an imbalanced dataset, where the non-stress class constitutes 75.17% and the stress class 24.83% of the dataset.

**Table 13:** Classification report of MLP classifier (hyper-parameterized).

**Class**	**Precision**	**Recall**	**f-Measure**
Non-stress	0.81	0.73	0.77
Stress	0.72	0.80	0.76
Accuracy	0.76		

### 5.3 Unsupervised learning architectures performance

We previously posited that the BIRCH algorithm provides advantages in terms of runtime, CPU utilization, and memory management. To validate this theoretical information, runtime and resource utilization experiments were conducted on a server equipped with an NVIDIA RTX A2000 GPU, 11th Gen Intel(R) Core(TM) i7-11850H @2.50GHz (16 Cores), and 32 GB RAM.

Upon examination of Table 14, it is evident that the BIRCH algorithm exhibits more efficient CPU and RAM utilization compared to other algorithms. Additionally, BIRCH demonstrated a shorter runtime than the others. The DBSCAN algorithm's attempt to handle even the smallest density regions was observed to result in longer runtimes and higher resource demands.

**Table 14:** Runtime and resource utilization.

**Algorithm**	**Runtime (ms)**	**CPU Util. (%)**	**RAM Util. (%)**
K-Means	85,809	0.77	0.12
DBSCAN	10,1286	0.81	0.14
BIRCH	65,052	0.73	0.10

To make a final decision, the accuracy metric of the algorithms was calculated using the data samples labeled by the clustering algorithms and the existing ground truth samples. The accuracy scores are presented in Table 15. The K-means algorithm achieved a higher accuracy score (73%) than BIRCH and DBSCAN, successfully clustering stress and non-stress samples. It's important to note that K-means assumes convex clusters with similar sizes, and its performance may vary in datasets with more features.

**Table 15:** Accuracy results of SL & SSL & UL architectures.

**Model**	**Hyp. tuning**	**Accuracy**
**Supervised learning results**		
LSTM	Yes	90%
CNN-LSTM	Yes	91%
**Semi-supervised learning results**		
Label propagation (RF classifier)	Yes	**77%**
Autoencoder (LR classifier-lbfgs)	No	61%
Autoencoder (LR classifier-saga)	No	63%
Autoencoder (RF classifier)	Yes	68%
Autoencoder (MLP classifier)	No	68%
Autoencoder (MLP classifier)	Yes	**76%**
**Unsupervised learning results**		
K-Means	Yes	73%
BIRCH	Yes	70%
DBSCAN	Yes	56%

The bold values indicate the highest accuracy performance.

BIRCH performed closely to the K-means result and demonstrated a crucial trade-off in terms of runtime and resource usage, making it advantageous for high-dimensional datasets. The lower accuracy score of DBSCAN suggests that it struggles with variable density regions in the dataset, resulting in almost random predictions.

### 5.4 Continuous stress level prediction

In the exploration of continuous stress level prediction using regressors, an analysis was conducted on binary stress and relax state classification, and additional focus was given to continuous prediction of stress levels. The ambulatory PSS-5, with scores ranging from 0 to 30, was categorized into three levels based on the guidelines from the PSS-14 official website (PSS, 2020). Scores were then assigned as 1, 2, and 3.

Regressors were applied to measure mean square errors (MSE) and mean absolute errors (MAE) for Heart Activity and Electrodermal Activity modalities separately. Traditional algorithms, including kNN, SVM, Random Forest, MLP, and Linear Regression, were tested after extracting handcrafted features from both modalities as outlined in the previous study (Can et al., 2019b). Hyperparameter optimization was performed with the following parameters: kNN (*k* = 6), SVM (Regularization parameter = 1.0, epsilon = 0.05, kernel = “rbf”), Random Forest (max_depth=2, random_state = 0, number_of_trees = 100), and MLP (activation = “tanh,” hidden_layer_sizes = (50, 100, 50), max_iter = 5,000). Additionally, LSTM and CNN-LSTM algorithms were tested.

The results are summarized in Table 16, where an MSE score of around 0.3 was achieved with the EDA signal. The regression codes have been included in the newly created repository.

**Table 16:** Performance comparison of classifiers for stress level regression from 1 to 3.

**Method**	**HA - MSE**	**HA - MAE**	**EDA - MSE**	**EDA - MAE**
Random Forest	0.39	0.53	0.33	0.48
MLP	0.38	0.54	0.344	0.464
SVM	0.40	0.51	0.36	0.41
kNN	0.47	0.49	0.41	0.51
Linear Regression	0.41	0.55	0.33	0.48
LSTM	0.36	0.47	0.28	0.43
LSTM-CNN	0.34	0.44	0.25	0.41

Heart activity (HA) from PPG data and electrodermal activity (EDA) from GSR sensors evaluated separately. Mean square error (MSE) and mean absolute error (MAE) were calculated.

## 6 Conclusion

In conclusion, this study focused on the semi-supervised classification of mental stress in daily life, aiming to address the labeling problem of sensory data. We designed LP and deep autoencoder models and compared their performance with existing SL (LSTM, CNN-LSTM) and UL (K-means, BIRCH, DBSCAN) algorithms. Specifically, for time-series data, we used the time-series split method instead of the conventional train-test split model in the cross-validation phase to accurately evaluate the performance of our LSTM and CNN-LSTM models.

This study is the first to investigate semi-supervised mental stress classification using daily life physiological data with a graph-based label propagation algorithm and deep autoencoder model. The accuracy scores of our models are presented in Table 15. We also provide a comparison with daily life stress recognition studies (see Table 17). Since most of the studies use different datasets, one can not infer directly the success of a technique over others. They were presented to give the reader a sense about the performance of state-of-the-art stress recognition techniques in daily life. While SL architectures outperformed in terms of performance, as they benefit from having ground truth labels beforehand, the results of our label propagation algorithm, which was designed with very limited labeled data, are also promising. The LP algorithm achieved precision, accuracy, and f-measure performance metrics that were close to those of the SL models. The deep autoencoder, initially categorized under UL, was utilized in our study from a semi-supervised learning perspective. Such an approach using raw daily life data has not been explored in the literature.

**Table 17:** Performance comparison of daily life stress recognition methods.

**References**	**Dataset**	**Type of learning**	**Method**	**Accuracy**
Yu and Sano (2022)	SMILE, TILES CrossCheck	Semi-supervised learning	LSTM-autoencoder	64%–70%
Yu and Sano (2023)	SMILE, TILES	Semi-supervised learning	LSTM-Autoencoder	63.44%
This study (2023)	Lab_to_Daily	Semi-supervised learning	Label Propagation	77%
This study (2023)	Lab_to_Daily	Semi-supervised learning	AutoEncoder	76%
Inoue (2018)	Local	Supervised learning	kNN, SVM, Decision tree	85.40%
Gjoreski et al. (2016)	Local	Supervised learning	Random forest	92%
Can and André (2023)	Lab_to_Daily	Supervised learning	LSTM, GRU, CNN-LSTM, 1D CNN	95%

However, when examining the performance of the deep autoencoder with a logistic regression classifier, it achieved a lower accuracy score compared to some clustering algorithms. One possible reason for this discrepancy is imperfect decoding, where the lossy reconstruction phase may have resulted in decreased performance or the augmented dataset obtained with the deep autoencoder may not have been compatible with the subsequent classifier. Consequently, we conducted experiments with different classifiers after the deep autoencoder model. Initially, we trained an RF classifier with the same parameters to compare it with the label propagation algorithm. The deep autoencoder, when trained with the RF classifier, exhibited a 5% improvement over the LR classifier, yet it still performed nearly 10% lower than the label propagation algorithm. Nevertheless, the results were closer to those obtained with clustering algorithms.

Based on these findings, we decided to test the autoencoder model with a more advanced classifier. Thus, we employed the MLP classifier and obtained the best parameters through hyper-parameter tuning. As a result, the overall accuracy score approached that of the LP algorithm. In summary, the performance results demonstrate the superiority of SSL architectures over UL architectures. Moreover, while LSTM and CNN-LSTM models achieved high performance, the labeling burden cannot be disregarded. Therefore, utilizing SSL architectures to achieve results with minimal labels would be more advantageous. However, the study is not without limitations. We used one of the largest daily life multimodal physiological datasets for stress recognition (around 1,000 h) but the number of participants is still relatively small.

Since most of the data is recorded from a similar age group (college students aged between 20 and 25), there is a likely bias leading classifiers to perform better for that age group and it limits the generalizability of findings. Future work should involve a larger and more representative population. We also relied on subjective reports as the ground truth for stress levels in the wild. However, unfortunately, they are the only alternative to annotate the data in the wild. They have several problems such as subjectivity, they can change from person to person, people can try to hide their real emotions, people can forget stressful events at the end of the session and participants might not give importance to the experiment. Having said that, more context information such as the number of people nearby, location of the participant, activity type (eating, working, presentation, etc.), and physical activity intensity can improve the reliability of the self-report ground truth. In future research, hybrid models can be constructed, incorporating CNN or LSTM layers, particularly on the autoencoder side, to potentially yield improved results. A promising research area is deep clustering in UL, where researchers aim to enhance the effectiveness of clustering algorithms by leveraging neural networks to extract data features.

## Data availability statement

The datasets presented in this study can be found in online repositories. The names of the repository/repositories and accession number(s) can be found in the article/supplementary material.

## Ethics statement

The procedure of the methodology used in this study was approved by the Institutional Review Board for Research with Human Subjects of Bogazici University with the approval number 2018/16. The studies were conducted in accordance with the local legislation and institutional requirements. The participants provided their written informed consent to participate in this study. Written informed consent was obtained from the individual(s) for the publication of any potentially identifiable images or data included in this article.

## Author contributions

OB: Conceptualization, Data curation, Investigation, Methodology, Software, Validation, Visualization, Writing – original draft, Writing – review & editing. YC: Formal analysis, Resources, Supervision, Validation, Writing – review & editing. EA: Formal analysis, Funding acquisition, Project administration, Resources, Supervision, Writing – review & editing. CE: Formal analysis, Project administration, Resources, Supervision, Validation, Writing – review & editing.
